# Supplementary material for: Comparative analysis of chloroplast genomes for five Dicliptera species (Acanthaceae): molecular structure, phylogenetic relationships, and adaptive evolution
Source: PeerJ. 2020 Feb 6;8:e8450. doi: 10.7717/peerj.8450 (PMC7007973; doi:10.7717/peerj.8450)
Supplement: Table S1 [file peerj-08-8450-s001.docx]

**Table S1.** Summary of 10 Acanthaceae chloroplast genome Genbank accession numbers.

| **Species name** | **Genbank accession numbers** |
| --- | --- |
| *Andrographis paniculata* | NC_022451 |
| *Ruellia breedlovei* | KP300014 |
| *Strobilanthes cusia* | MG874806 |
| *Echinacanthus attenuatus* | NC_039762.1 |
| *Echinacanthus longipes* | NC_039761.1 |
| *Echinacanthus longzhouensis* | NC_039678.1 |
| *Echinacanthus lofouensis* | NC_035876.1 |
| *Aphelandra knappiae* | MH909777 |
| *Justicia leptostachya* | MK389502 |
| *Clinacanthus nutans* | MH778102.1 |
