# Supplementary material for: Comparative analysis of chloroplast genomes for five Dicliptera species (Acanthaceae): molecular structure, phylogenetic relationships, and adaptive evolution
Source: PeerJ. 2020 Feb 6;8:e8450. doi: 10.7717/peerj.8450 (PMC7007973; doi:10.7717/peerj.8450)
Supplement: Table S2 [file peerj-08-8450-s002.docx]

**Table S2.** List of genes in the chloroplast genome of five *Dicliptera* species.

| Category | Gene groups | Name of genes |
| --- | --- | --- |
| Self-replication(59) | Large subunit of ribosomal proteins | *rpl2*×2, *rpl14*, *rpl16*, *rpl20*, *rpl22*, *rpl23*×2, *rpl32*, *rpl33*, *rpl36* |
|  | Small subunit of ribosomal porteins | *rps2*, *rps3*, *rps4*, *rps7*×2, *rps8*, *rps11*, *rps12*, *rps14*, *rps15*, *rps16*, *rps18*, *rps19* |
|  | RNA polymerase | *rpoA*, *rpoB*, *rpoC1*, *rpoC2* |
|  | Ribosomal RNA gene | *rrn4.5*×2, *rrn5*×2, *rrn16*×2, *rrn23*×2 |
|  | Transfer RNA genes | *trnA*-UGC×2, *trnC*-GCA, *trnD*-GUC, *trnE*-UUC, *trnF*-GAA, *trnfM*-CAU, *trnG-*GCC, *trnG*-UCC, *trnH-GUG*, *trnI*-CAU×2, *trnI*-GAU×2, *trnK*-UUU, *trnL-*CAA×2, *trnL*-UAA, *trnL*-UAG, *trnM*-CAU, *trnN*-GUU×2, *trnP*-UGG, *trnQ*-UUG, *trnR-*ACG×2, *trnR*-UCU, *trnS*-GCU, *trnS*-GGA, *trnS*-UGA, *trnT*-GGU, *trnT*-UGU, *trnV-*GAC×2, *trnV-*UAC, *trnW-*CCA, *trnY-*GUA |
|  | Translational initiation factor | *infA* |
| Photosynthesis(46) | Subunits of ATP synthase | *atpA*, *atpB*, *atpE*, *atpF*, *atpH*, *atpI* |
|  | Subunits of Photosystem Ⅰ | *psaA*, *psaB*, *psaC*, *psaI*, *psaJ*, *ycf3*, *ycf4* |
|  | Subunits of Photosystem Ⅱ | *psbA*, *psbB*, *psbC*, *psbD*, *psbE*, *psbF*, *psbH*, *psbI*, *psbJ*, *psbK*, *psbL*, *psbM*, *psbN*, *psbT*, *psbZ* |
|  | Subunits of cytochrome b/f complex | *petA*, *petB*, *petD*, *petG*, *petL*, *petN* |
|  | Subunits of rubisco | *rbcL* |
|  | Subunits of NADH-dehydrogenase | *ndhA*, *ndhB*×2, *ndhC*, *ndhD*, *ndhE*, *ndhF*, *ndhG*, *ndhH*, *ndhI* , *ndhJ*, *ndhK* |
| Other genes(9) | Subunit of acetyl-CoAcarboxylase | *accD* |
|  | C-type cytochorome synthesis gene | *ccsA* |
|  | Envelope membrane protein | *cemA* |
|  | ATP-dependent Protease | *clpP* |
|  | Maturase K | *matK* |
|  | Component of TIC complex | *ycf1* |
|  | Genes of unknown function | *ycf2*×2, *ycf15*×2 |
