# Supplementary material for: Comparative analysis of chloroplast genomes for five Dicliptera species (Acanthaceae): molecular structure, phylogenetic relationships, and adaptive evolution
Source: PeerJ. 2020 Feb 6;8:e8450. doi: 10.7717/peerj.8450 (PMC7007973; doi:10.7717/peerj.8450)
Supplement: Table S3 [file peerj-08-8450-s003.docx]

**Table S3.** nucleotide diversity (Pi) values of the regions in *Dicliptera.*

| **Region** | **Pi** | **Region** | **Pi** |
| --- | --- | --- | --- |
| *trnH-*GUG | 0.00000 | *trnH-*GUG*-psbA* | 0.00267 |
| *psbA* | 0.00038 | *psbA-trnK-*UUU | 0.00000 |
| *trnK-*UUU | 0.00064 | *trnK-*UUU*-rps16* | 0.00000 |
| *matK* | 0.00107 | *rps16-trnQ-*UUG | 0.00112 |
| *rps16* | 0.00103 | *trnQ-*UUG*-psbK* | 0.00112 |
| *trnQ-*UUG | 0.00000 | *psbK-psbI* | 0.00093 |
| *psbK* | 0.00000 | *psbI-trnS-*GCU | 0.00000 |
| *psbI* | 0.00000 | *trnS-*GCU*-trnG-*UCC | 0.00118 |
| *trnS-*GCU | 0.00000 | *trnG-*UCC*-trnR-*UCU | 0.00000 |
| *trnG-*UCC | 0.00000 | *trnR-*UCU*-atpA* | 0.00000 |
| *trnR-*UCU | 0.00000 | *atpA-atpF* | 0.00036 |
| *atpA* | 0.00000 | *atpF-atpH* | 0.00000 |
| *atpF* | 0.00000 | *atpH-atpI* | 0.00051 |
| *atpH* | 0.00000 | *atpI-rps2* | 0.00000 |
| *atpI* | 0.00000 | *rps2-rpoC2* | 0.00000 |
| *rps2* | 0.00000 | *rpoC2-rpoC1* | 0.00000 |
| *rpoC2* | 0.00029 | *rpoC1-rpoB* | 0.00000 |
| *rpoC1* | 0.00028 | *rpoB-trnC-*GCA | 0.00067 |
| *rpoB* | 0.00000 | *trnC-*GCA*-petN* | 0.00000 |
| *trnC-*GCA | 0.00000 | *petN-psbM* | 0.00783 |
| *petN* | 0.00000 | *psbM-trnD-*GUC | 0.00000 |
| *psbM* | 0.00000 | *trnD-*GUC*-trnY-*GUA | 0.00000 |
| *trnD-*GUC | 0.00000 | *trnY-*GUA*-trnE-*UUC | 0.00526 |
| *trnY-*GUA | 0.00000 | *trnE-*UUC*-trnT-*GGU | 0.00193 |
| *trnE-*UUC | 0.00000 | *trnT-*GGU*-psbD* | 0.00035 |
| *trnT-*GGU | 0.00000 | *psbC-trnS-*UGA | 0.00000 |
| *psbD* | 0.00038 | *trnS-*UGA*-psbZ* | 0.00000 |
| *psbC* | 0.00056 | *psbZ-trnG-*GCC | 0.00697 |
| *trnS-*UGA | 0.00000 | *trnG-*GCC*-trnfM-*CAU | 0.00284 |
| *psbZ* | 0.00000 | *trnfM-*CAU*-rps14* | 0.00000 |
| *trnG-*GCC | 0.00571 | *rps14-psaB* | 0.00000 |
| *trnfM-*CAU | 0.00000 | *psaB-psaA* | 0.00000 |
| *rps14* | 0.00000 | *psaA-ycf3* | 0.00000 |
| *psaB* | 0.00000 | *ycf3-trnS-*GGA | 0.00000 |
| *psaA* | 0.00000 | *trnS-*GGA*-rps4* | 0.00000 |
| *ycf3* | 0.00042 | *rps4-trnL-*UUA | 0.02230 |
| *trnS-*GGA | 0.00000 | *trnL-*UUA*-trnF-*GAA | 0.00000 |
| *rps4* | 0.00000 | *trnF-*GAA*-ndhJ* | 0.00140 |
| *trnL-*UUA | 0.00073 | *ndhJ-ndhK* | 0.00000 |
| *trnF-*GAA | 0.00000 | *ndhK-ndhC* | 0.00000 |
| *ndhJ* | 0.00000 | *ndhC-trnV-*UAC | 0.00000 |
| *ndhK* | 0.00000 | *trnV-*UAC*-trnM-*CAU | 0.00000 |
| *ndhC* | 0.00000 | *trnM-*CAU*-atpE* | 0.00000 |
| *trnV-*UAC | 0.00060 | *atpB-rbcL* | 0.00000 |
| *trnM-*CAU | 0.00000 | *rbcL-accD* | 0.00163 |
| *atpE* | 0.00000 | *accD-psaI* | 0.00057 |
| *atpB* | 0.00000 | *psaI-ycf4* | 0.00000 |
| *rbcL* | 0.00000 | *ycf4-cemA* | 0.00107 |
| *accD* | 0.00027 | *cemA-petA* | 0.00000 |
| *psaI* | 0.00364 | *petA-psbJ* | 0.00000 |
| *ycf4* | 0.00000 | *psbJ-psbL* | 0.00000 |
| *cemA* | 0.00117 | *psbL-psbF* | 0.00000 |
| *petA* | 0.00000 | *psbF-psbE* | 0.00000 |
| *psbJ* | 0.00000 | *psbE-petL* | 0.00046 |
| *psbL* | 0.00000 | *petL-petG* | 0.00000 |
| *psbF* | 0.00000 | *petG-trnW-*CCA | 0.00000 |
| *psbE* | 0.00159 | *trnW-*CCA*-trnP-*UGG | 0.00000 |
| *petL* | 0.00000 | *trnP-*UGG*-psaJ* | 0.00000 |
| *petG* | 0.00000 | *psaJ-rpl33* | 0.00000 |
| *trnW-*CCA | 0.00000 | *rpl33-rps18* | 0.00000 |
| *trnP-*UGG | 0.00000 | *rps18-rpl20* | 0.00000 |
| *psaJ* | 0.00000 | *clpP-psbB* | 0.00000 |
| *rpl33* | 0.00000 | *psbB-psbT* | 0.00000 |
| *rps18* | 0.00131 | *psbT-psbN* | 0.00000 |
| *rpl20* | 0.00000 | *psbN-psbH* | 0.00000 |
| *clpP* | 0.00062 | *psbH-petB* | 0.00000 |
| *psbB* | 0.00000 | *petB-petD* | 0.00196 |
| *psbT* | 0.00000 | *petD-rpoA* | 0.00000 |
| *psbN* | 0.00000 | *rpoA-rps11* | 0.00000 |
| *psbH* | 0.00000 | *rps11-rpl36* | 0.00000 |
| *petB* | 0.00059 | *rpl36-infA* | 0.00000 |
| *petD* | 0.00066 | *infA-rps8* | 0.00000 |
| *rpoA* | 0.00059 | *rps8-rpl14* | 0.00000 |
| *rps11* | 0.00000 | *rpl14-rpl16* | 0.00000 |
| *rpl36* | 0.00000 | *rpl16-rps3* | 0.00000 |
| *infA* | 0.00169 | *rpl22-rps19* | 0.00000 |
| *rps8* | 0.00099 | *rps19-rpl2* | 0.00000 |
| *rpl14* | 0.00133 | *rpl2-rpl23* | 0.00000 |
| *rpl16* | 0.00028 | *rpl23-trnH-*CAU | 0.00000 |
| *rps3* | 0.00000 | *trnH-*CAU*-ycf2* | 0.00000 |
| *rpl22* | 0.00000 | *ycf2-ycf15* | 0.00000 |
| *rps19* | 0.00143 | *ycf15-trnL-*CAA | 0.00000 |
| *rpl2* | 0.00000 | *trnL-*CAA*-ndhB* | 0.00000 |
| *rpl23* | 0.00000 | *ndhB-rps7* | 0.00000 |
| *trnH-*CAU | 0.00000 | *rps7-trnV-*UAC | 0.00017 |
| *ycf2* | 0.00006 | *trnV-*GAC*-rrn16* | 0.00000 |
| *ycf15* | 0.00000 | *rrn16-trnI-*GAU | 0.00000 |
| *trnL-*CAA | 0.00000 | *trnI-*GAU*-trnA-*UGC | 0.00000 |
| *ndhB* | 0.00018 | *trnA-*UGC*-rrn23* | 0.00000 |
| *rps7* | 0.00086 | *rrn23-rrn4.5* | 0.00000 |
| *trnV-*GAC | 0.00000 | *rrn4.5-rrn5* | 0.00000 |
| *rrn16* | 0.00000 | *rrn5-trnR-*ACG | 0.00000 |
| *trnI-*GAU | 0.00000 | *trnR-*ACG*-trnN-*GUU | 0.00000 |
| *trnA-*UGC | 0.00000 | *trnN-*GUU*-ndhF* | 0.00000 |
| *rrn23* | 0.00000 | *ndhF-rpl32* | 0.00000 |
| *rrn4.5* | 0.00000 | *rpl32-trnL-*UAG | 0.00000 |
| *rrn5* | 0.00000 | *trnL-*UAG*-ccsA* | 0.00000 |
| *trnR-*ACG | 0.00000 | *ccsA-ndhD* | 0.00000 |
| *trnN-*GUU | 0.00000 | *ndhD-psaC* | 0.00000 |
| *ndhF* | 0.00068 | *psaC-ndhE* | 0.00000 |
| *rpl32* | 0.00000 | *ndhE-ndhG* | 0.00000 |
| *trnL-*UAG | 0.00000 | *ndhG-ndhI* | 0.00118 |
| *ccsA* | 0.00000 | *ndhI-ndhA* | 0.00000 |
| *ndhD* | 0.00055 | *ndhA-ndhH* | 0.00034 |
| *psaC* | 0.00000 | *ndhH-rps15* | 0.00000 |
| *ndhE* | 0.00000 | *rps15-ycf1* | 0.00000 |
| *ndhG* | 0.00151 | *ndhH* | 0.00034 |
| *ndhI* | 0.00000 | *rps15* | 0.00000 |
| *ndhA* | 0.00019 | *ycf1* | 0.00109 |
