# Supplementary material for: Comparative analysis of chloroplast genomes for five Dicliptera species (Acanthaceae): molecular structure, phylogenetic relationships, and adaptive evolution
Source: PeerJ. 2020 Feb 6;8:e8450. doi: 10.7717/peerj.8450 (PMC7007973; doi:10.7717/peerj.8450)
Supplement: Table S4 [file peerj-08-8450-s004.docx]

**Table S4.** Codon frequencies and relative synonymous codon usage (RSCU) values of the cp genomes of *D. acuminata, D. peruviana, D. montana, D. ruiziana and D. mucronata*.

| **Species** | **Amino Acid** | **Codon** | **Count** | **RSCU** | **tRNA** |
| --- | --- | --- | --- | --- | --- |
| *D. acuminata* | Phe | UUU | 985 | **1.31** |  |
|  |  | UUC | 520 | 0.69 | trnF-GAA |
|  | Leu | UUA | 869 | **1.85** | trnL-UAA |
|  |  | UUG | 589 | **1.25** | trnL-CAA |
|  |  | CUU | 597 | **1.27** |  |
|  |  | CUC | 186 | 0.4 |  |
|  |  | CUA | 387 | 0.82 | trnL-UAG |
|  |  | CUG | 197 | 0.42 |  |
|  | Ile | AUU | 1106 | **1.5** |  |
|  |  | AUC | 456 | 0.62 | trnI-GAU |
|  |  | AUA | 646 | 0.88 |  |
|  | Met | AUG | 597 | 1 | trnfM-CAU, trnI-CAU, trnM-CAU |
|  | Val | GUU | 531 | **1.5** |  |
|  |  | GUC | 167 | 0.47 | trnV-GAC |
|  |  | GUA | 524 | **1.48** | trnV-UAC |
|  |  | GUG | 195 | 0.55 |  |
|  | Ser | UCU | 581 | **1.71** |  |
|  |  | UCC | 319 | 0.94 | trnS-GGA |
|  |  | UCA | 403 | **1.18** | trnS-UGA |
|  |  | UCG | 220 | 0.65 |  |
|  | Pro | CCU | 411 | **1.51** |  |
|  |  | CCC | 214 | 0.79 |  |
|  |  | CCA | 294 | **1.08** | trnP-UGG |
|  |  | CCG | 168 | 0.62 |  |
|  | Thr | ACU | 542 | **1.61** |  |
|  |  | ACC | 242 | 0.72 | trnT-GGU |
|  |  | ACA | 399 | **1.19** | trnT-UGU |
|  |  | ACG | 161 | 0.48 |  |
|  | Ala | GCU | 628 | **1.79** |  |
|  |  | GCC | 230 | 0.65 |  |
|  |  | GCA | 397 | **1.13** | trnA-UGC |
|  |  | GCG | 152 | 0.43 |  |
|  | Tyr | UAU | 757 | **1.61** |  |
|  |  | UAC | 182 | 0.39 | trnY-GUA |
|  | TER | UAA | 45 | **1.55** |  |
|  |  | UAG | 26 | 0.9 |  |
|  | His | CAU | 470 | **1.52** |  |
|  |  | CAC | 147 | 0.48 | trnH-GUG |
|  | Gln | CAA | 721 | **1.53** | trnQ-UUG |
|  |  | CAG | 219 | 0.47 |  |
|  | Asn | AAU | 956 | **1.53** |  |
|  |  | AAC | 297 | 0.47 | trnN-GUU |
|  | Lys | AAA | 1065 | **1.49** | trnK-UUU |
|  |  | AAG | 368 | 0.51 |  |
|  | Asp | GAU | 858 | **1.65** |  |
|  |  | GAC | 183 | 0.35 | trnD-GUC |
|  | Glu | GAA | 983 | **1.47** | trnE-UUC |
|  |  | GAG | 352 | 0.53 |  |
|  | Cys | UGU | 226 | **1.48** |  |
|  |  | UGC | 79 | 0.52 | trnC-GCA |
|  | TER | UGA | 16 | 0.55 |  |
|  | Trp | UGG | 460 | 1 | trnW-CCA |
|  | Arg | CGU | 325 | **1.2** | trnR-ACG |
|  |  | CGC | 108 | 0.4 |  |
|  |  | CGA | 382 | **1.42** |  |
|  |  | CGG | 146 | 0.54 |  |
|  | Ser | AGU | 416 | **1.22** |  |
|  |  | AGC | 105 | 0.31 | trnS-GCU |
|  | Arg | AGA | 477 | **1.77** | trnR-UCU |
|  |  | AGG | 181 | 0.67 |  |
|  | Gly | GGU | 573 | **1.29** |  |
|  |  | GGC | 166 | 0.37 | trnG-GCC |
|  |  | GGA | 697 | **1.57** | trnG-UCC |
|  |  | GGG | 339 | 0.76 |  |
| *D. peruviana* | Phe | UUU | 985 | **1.31** |  |
|  |  | UUC | 520 | 0.69 | trnF-GAA |
|  | Leu | UUA | 871 | **1.85** | trnL-UAA |
|  |  | UUG | 587 | **1.25** | trnL-CAA |
|  |  | CUU | 597 | **1.27** |  |
|  |  | CUC | 187 | 0.4 |  |
|  |  | CUA | 386 | 0.82 | trnL-UAG |
|  |  | CUG | 197 | 0.42 |  |
|  | Ile | AUU | 1107 | **1.5** |  |
|  |  | AUC | 455 | 0.62 | trnI-GAU |
|  |  | AUA | 645 | 0.88 |  |
|  | Met | AUG | 597 | 1 | trnfM-CAU, trnI-CAU, trnM-CAU |
|  | Val | GUU | 531 | **1.5** |  |
|  |  | GUC | 167 | 0.47 | trnV-GAC |
|  |  | GUA | 524 | **1.48** | trnV-UAC |
|  |  | GUG | 195 | 0.55 |  |
|  | Ser | UCU | 580 | **1.7** |  |
|  |  | UCC | 320 | 0.94 | trnS-GGA |
|  |  | UCA | 403 | **1.18** | trnS-UGA |
|  |  | UCG | 222 | 0.65 |  |
|  | Pro | CCU | 411 | **1.51** |  |
|  |  | CCC | 214 | 0.79 |  |
|  |  | CCA | 294 | **1.08** | trnP-UGG |
|  |  | CCG | 168 | 0.62 |  |
|  | Thr | ACU | 541 | **1.61** |  |
|  |  | ACC | 242 | 0.72 | trnT-GGU |
|  |  | ACA | 399 | **1.19** | trnT-UGU |
|  |  | ACG | 161 | 0.48 |  |
|  | Ala | GCU | 629 | **1.78** |  |
|  |  | GCC | 230 | 0.65 |  |
|  |  | GCA | 397 | **1.13** | trnA-UGC |
|  |  | GCG | 154 | 0.44 |  |
|  | Tyr | UAU | 757 | **1.61** |  |
|  |  | UAC | 183 | 0.39 | trnY-GUA |
|  | TER | UAA | 45 | **1.55** |  |
|  |  | UAG | 26 | 0.9 |  |
|  | His | CAU | 470 | **1.52** |  |
|  |  | CAC | 147 | 0.48 | trnH-GUG |
|  | Gln | CAA | 721 | **1.53** | trnQ-UUG |
|  |  | CAG | 219 | 0.47 |  |
|  | Asn | AAU | 956 | **1.52** |  |
|  |  | AAC | 298 | 0.48 | trnN-GUU |
|  | Lys | AAA | 1065 | **1.49** | trnK-UUU |
|  |  | AAG | 368 | 0.51 |  |
|  | Asp | GAU | 858 | **1.65** |  |
|  |  | GAC | 182 | 0.35 | trnD-GUC |
|  | Glu | GAA | 983 | **1.47** | trnE-UUC |
|  |  | GAG | 352 | 0.53 |  |
|  | Cys | UGU | 226 | **1.49** |  |
|  |  | UGC | 77 | 0.51 | trnC-GCA |
|  | TER | UGA | 16 | 0.55 |  |
|  | Trp | UGG | 460 | 1 | trnW-CCA |
|  | Arg | CGU | 325 | **1.2** | trnR-ACG |
|  |  | CGC | 109 | 0.4 |  |
|  |  | CGA | 382 | **1.42** |  |
|  |  | CGG | 144 | 0.53 |  |
|  | Ser | AGU | 416 | **1.22** |  |
|  |  | AGC | 105 | 0.31 | trnS-GCU |
|  | Arg | AGA | 478 | **1.77** | trnR-UCU |
|  |  | AGG | 181 | 0.67 |  |
|  | Gly | GGU | 574 | **1.29** |  |
|  |  | GGC | 166 | 0.37 | trnG-GCC |
|  |  | GGA | 697 | **1.57** | trnG-UCC |
|  |  | GGG | 336 | 0.76 |  |
| *D. montana* | Phe | UUU | 984 | **1.31** |  |
|  |  | UUC | 520 | 0.69 | trnF-GAA |
|  | Leu | UUA | 871 | **1.85** | trnL-UAA |
|  |  | UUG | 589 | **1.25** | trnL-CAA |
|  |  | CUU | 597 | **1.27** |  |
|  |  | CUC | 186 | 0.39 |  |
|  |  | CUA | 387 | 0.82 | trnL-UAG |
|  |  | CUG | 197 | 0.42 |  |
|  | Ile | AUU | 1106 | **1.5** |  |
|  |  | AUC | 456 | 0.62 | trnI-GAU |
|  |  | AUA | 646 | 0.88 |  |
|  | Met | AUG | 597 | 1 | trnfM-CAU, trnI-CAU, trnM-CAU |
|  | Val | GUU | 531 | **1.5** |  |
|  |  | GUC | 167 | 0.47 | trnV-GAC |
|  |  | GUA | 524 | **1.48** | trnV-UAC |
|  |  | GUG | 195 | 0.55 |  |
|  | Ser | UCU | 581 | **1.7** |  |
|  |  | UCC | 319 | 0.94 | trnS-GGA |
|  |  | UCA | 404 | **1.19** | trnS-UGA |
|  |  | UCG | 220 | 0.65 |  |
|  | Pro | CCU | 411 | **1.51** |  |
|  |  | CCC | 214 | 0.79 |  |
|  |  | CCA | 293 | **1.08** | trnP-UGG |
|  |  | CCG | 169 | 0.62 |  |
|  | Thr | ACU | 542 | **1.61** |  |
|  |  | ACC | 242 | 0.72 | trnT-GGU |
|  |  | ACA | 399 | **1.19** | trnT-UGU |
|  |  | ACG | 161 | 0.48 |  |
|  | Ala | GCU | 628 | **1.78** |  |
|  |  | GCC | 230 | 0.65 |  |
|  |  | GCA | 397 | **1.13** | trnA-UGC |
|  |  | GCG | 154 | 0.44 |  |
|  | Tyr | UAU | 758 | **1.61** |  |
|  |  | UAC | 183 | 0.39 | trnY-GUA |
|  | TER | UAA | 45 | **1.55** |  |
|  |  | UAG | 26 | 0.9 |  |
|  | His | CAU | 469 | **1.52** |  |
|  |  | CAC | 147 | 0.48 | trnH-GUG |
|  | Gln | CAA | 721 | **1.53** | trnQ-UUG |
|  |  | CAG | 219 | 0.47 |  |
|  | Asn | AAU | 956 | **1.52** |  |
|  |  | AAC | 298 | 0.48 | trnN-GUU |
|  | Lys | AAA | 1065 | **1.49** | trnK-UUU |
|  |  | AAG | 368 | 0.51 |  |
|  | Asp | GAU | 858 | **1.65** |  |
|  |  | GAC | 182 | 0.35 | trnD-GUC |
|  | Glu | GAA | 983 | **1.47** | trnE-UUC |
|  |  | GAG | 352 | 0.53 |  |
|  | Cys | UGU | 226 | **1.49** |  |
|  |  | UGC | 77 | 0.51 | trnC-GCA |
|  | TER | UGA | 16 | 0.55 |  |
|  | Trp | UGG | 460 | 1 | trnW-CCA |
|  | Arg | CGU | 325 | **1.21** | trnR-ACG |
|  |  | CGC | 108 | 0.4 |  |
|  |  | CGA | 382 | **1.42** |  |
|  |  | CGG | 145 | 0.54 |  |
|  | Ser | AGU | 416 | **1.22** |  |
|  |  | AGC | 105 | 0.31 | trnS-GCU |
|  | Arg | AGA | 477 | **1.77** | trnR-UCU |
|  |  | AGG | 181 | 0.67 |  |
|  | Gly | GGU | 574 | **1.29** |  |
|  |  | GGC | 166 | 0.37 | trnG-GCC |
|  |  | GGA | 697 | **1.57** | trnG-UCC |
|  |  | GGG | 336 | 0.76 |  |
| *D. ruiziana* | Phe | UUU | 985 | **1.31** |  |
|  |  | UUC | 520 | 0.69 | trnF-GAA |
|  | Leu | UUA | 872 | **1.85** | trnL-UAA |
|  |  | UUG | 589 | **1.25** | trnL-CAA |
|  |  | CUU | 597 | **1.27** |  |
|  |  | CUC | 186 | 0.39 |  |
|  |  | CUA | 387 | 0.82 | trnL-UAG |
|  |  | CUG | 197 | 0.42 |  |
|  | Ile | AUU | 1107 | **1.5** |  |
|  |  | AUC | 455 | 0.62 | trnI-GAU |
|  |  | AUA | 646 | 0.88 |  |
|  | Met | AUG | 597 | 1 | trnfM-CAU, trnI-CAU, trnM-CAU |
|  | Val | GUU | 531 | **1.5** |  |
|  |  | GUC | 167 | 0.47 | trnV-GAC |
|  |  | GUA | 524 | **1.48** | trnV-UAC |
|  |  | GUG | 195 | 0.55 |  |
|  | Ser | UCU | 581 | **1.7** |  |
|  |  | UCC | 320 | 0.94 | trnS-GGA |
|  |  | UCA | 404 | **1.18** | trnS-UGA |
|  |  | UCG | 220 | 0.65 |  |
|  | Pro | CCU | 411 | **1.51** |  |
|  |  | CCC | 213 | 0.78 |  |
|  |  | CCA | 293 | **1.08** | trnP-UGG |
|  |  | CCG | 169 | 0.62 |  |
|  | Thr | ACU | 542 | **1.61** |  |
|  |  | ACC | 242 | 0.72 | trnT-GGU |
|  |  | ACA | 399 | **1.19** | trnT-UGU |
|  |  | ACG | 161 | 0.48 |  |
|  | Ala | GCU | 627 | **1.78** |  |
|  |  | GCC | 231 | 0.66 |  |
|  |  | GCA | 397 | **1.13** | trnA-UGC |
|  |  | GCG | 154 | 0.44 |  |
|  | Tyr | UAU | 755 | **1.61** |  |
|  |  | UAC | 183 | 0.39 | trnY-GUA |
|  | TER | UAA | 45 | **1.55** |  |
|  |  | UAG | 26 | 0.9 |  |
|  | His | CAU | 470 | **1.52** |  |
|  |  | CAC | 147 | 0.48 | trnH-GUG |
|  | Gln | CAA | 723 | **1.54** | trnQ-UUG |
|  |  | CAG | 219 | 0.46 |  |
|  | Asn | AAU | 958 | **1.52** |  |
|  |  | AAC | 300 | 0.48 | trnN-GUU |
|  | Lys | AAA | 1067 | **1.49** | trnK-UUU |
|  |  | AAG | 370 | 0.51 |  |
|  | Asp | GAU | 858 | **1.65** |  |
|  |  | GAC | 182 | 0.35 | trnD-GUC |
|  | Glu | GAA | 985 | **1.47** | trnE-UUC |
|  |  | GAG | 352 | 0.53 |  |
|  | Cys | UGU | 228 | **1.5** |  |
|  |  | UGC | 77 | 0.5 | trnC-GCA |
|  | TER | UGA | 16 | 0.55 |  |
|  | Trp | UGG | 459 | 1 | trnW-CCA |
|  | Arg | CGU | 325 | **1.21** | trnR-ACG |
|  |  | CGC | 108 | 0.4 |  |
|  |  | CGA | 382 | **1.42** |  |
|  |  | CGG | 145 | 0.54 |  |
|  | Ser | AGU | 416 | **1.22** |  |
|  |  | AGC | 105 | 0.31 | trnS-GCU |
|  | Arg | AGA | 475 | **1.76** | trnR-UCU |
|  |  | AGG | 181 | 0.67 |  |
|  | Gly | GGU | 574 | **1.29** |  |
|  |  | GGC | 166 | 0.37 | trnG-GCC |
|  |  | GGA | 697 | **1.57** | trnG-UCC |
|  |  | GGG | 336 | 0.76 |  |
| *D. mucronata* | Phe | UUU | 1159 | **1.31** |  |
|  |  | UUC | 608 | 0.69 | trnF-GAA |
|  | Leu | UUA | 608 | **1.7** | trnL-UAA |
|  |  | UUG | 457 | **1.28** | trnL-CAA |
|  |  | CUU | 502 | **1.4** |  |
|  |  | CUC | 175 | 0.49 |  |
|  |  | CUA | 267 | 0.75 | trnL-UAG |
|  |  | CUG | 141 | 0.39 |  |
|  | Ile | AUU | 1026 | **1.58** |  |
|  |  | AUC | 503 | 0.78 | trnI-GAU |
|  |  | AUA | 413 | 0.64 |  |
|  | Met | AUG | 391 | 1 | trnfM-CAU, trnI-CAU, trnM-CAU |
|  | Val | GUU | 442 | **1.56** |  |
|  |  | GUC | 167 | 0.59 | trnV-GAC |
|  |  | GUA | 359 | **1.27** | trnV-UAC |
|  |  | GUG | 166 | 0.59 |  |
|  | Ser | UCU | 650 | **1.53** |  |
|  |  | UCC | 369 | 0.87 | trnS-GGA |
|  |  | UCA | 505 | **1.19** | trnS-UGA |
|  |  | UCG | 327 | 0.77 |  |
|  | Pro | CCU | 319 | **1.42** |  |
|  |  | CCC | 183 | 0.81 |  |
|  |  | CCA | 242 | **1.08** | trnP-UGG |
|  |  | CCG | 155 | 0.69 |  |
|  | Thr | ACU | 439 | **1.32** |  |
|  |  | ACC | 298 | 0.9 | trnT-GGU |
|  |  | ACA | 379 | **1.14** | trnT-UGU |
|  |  | ACG | 214 | 0.64 |  |
|  | Ala | GCU | 470 | **1.72** |  |
|  |  | GCC | 196 | 0.72 |  |
|  |  | GCA | 292 | **1.07** | trnA-UGC |
|  |  | GCG | 138 | 0.5 |  |
|  | Tyr | UAU | 840 | **1.44** |  |
|  |  | UAC | 330 | 0.56 | trnY-GUA |
|  | TER | UAA | 375 | **1.12** |  |
|  |  | UAG | 219 | 0.65 |  |
|  | His | CAU | 461 | **1.43** |  |
|  |  | CAC | 183 | 0.57 | trnH-GUG |
|  | Gln | CAA | 634 | **1.51** | trnQ-UUG |
|  |  | CAG | 206 | 0.49 |  |
|  | Asn | AAU | 940 | **1.41** |  |
|  |  | AAC | 392 | 0.59 | trnN-GUU |
|  | Lys | AAA | 1013 | **1.44** | trnK-UUU |
|  |  | AAG | 390 | 0.56 |  |
|  | Asp | GAU | 720 | **1.62** |  |
|  |  | GAC | 168 | 0.38 | trnD-GUC |
|  | Glu | GAA | 853 | **1.5** | trnE-UUC |
|  |  | GAG | 283 | 0.5 |  |
|  | Cys | UGU | 361 | **1.2** |  |
|  |  | UGC | 239 | 0.8 | trnC-GCA |
|  | TER | UGA | 414 | **1.23** |  |
|  | Trp | UGG | 523 | 1 | trnW-CCA |
|  | Arg | CGU | 226 | 0.75 | trnR-ACG |
|  |  | CGC | 118 | 0.39 |  |
|  |  | CGA | 364 | **1.21** |  |
|  |  | CGG | 164 | 0.55 |  |
|  | Ser | AGU | 422 | 0.99 |  |
|  |  | AGC | 280 | 0.66 | trnS-GCU |
|  | Arg | AGA | 608 | **2.03** | trnR-UCU |
|  |  | AGG | 318 | **1.06** |  |
|  | Gly | GGU | 450 | **1.1** |  |
|  |  | GGC | 209 | 0.51 | trnG-GCC |
|  |  | GGA | 588 | **1.44** | trnG-UCC |
|  |  | GGG | 388 | 0.95 |  |
