# Supplementary material for: Comparative analysis of chloroplast genomes for five Dicliptera species (Acanthaceae): molecular structure, phylogenetic relationships, and adaptive evolution
Source: PeerJ. 2020 Feb 6;8:e8450. doi: 10.7717/peerj.8450 (PMC7007973; doi:10.7717/peerj.8450)
Supplement: Table S5 [file peerj-08-8450-s005.docx]

**Table S5.** Simple sequence repeats (SSRs) in the cp genomes of the five *Dicliptera* species.

| **Species** | **Gene ID** | **SSR nr** | **SSR type** | **SSR size** | **start** | **end** |
| --- | --- | --- | --- | --- | --- | --- |
| *D. acuminata* | 1 | p3 | (AAT)4 | 12 | 4116 | 4127 |
|  | 2 | p4 | (ATTG)3 | 12 | 5403 | 5414 |
|  | 3 | p2 | (AT)6 | 12 | 7230 | 7241 |
|  | 4 | p1 | (T)10 | 10 | 7374 | 7383 |
|  | 5 | p1 | (A)11 | 11 | 7595 | 7605 |
|  | 6 | p1 | (T)10 | 10 | 8275 | 8284 |
|  | 7 | p1 | (A)10 | 10 | 9251 | 9260 |
|  | 8 | p1 | (T)13 | 13 | 9458 | 9470 |
|  | 9 | c | (A)12(T)10 | 93 | 11660 | 11752 |
|  | 10 | p1 | (A)10 | 10 | 14521 | 14530 |
|  | 11 | c | (A)12(A)10 | 43 | 15469 | 15511 |
|  | 12 | p2 | (TA)5 | 10 | 19076 | 19085 |
|  | 13 | p2 | (AT)5 | 10 | 20107 | 20116 |
|  | 14 | p1 | (T)12 | 12 | 31282 | 31293 |
|  | 15 | p3 | (TTC)4 | 12 | 34132 | 34143 |
|  | 16 | c | (T)13(G)11 | 24 | 35218 | 35241 |
|  | 17 | p1 | (A)10 | 10 | 41322 | 41331 |
|  | 18 | p1 | (T)10 | 10 | 42266 | 42275 |
|  | 19 | p1 | (T)10 | 10 | 45002 | 45011 |
|  | 20 | c | (ATAA)3(TA)6 | 62 | 45255 | 45316 |
|  | 21 | p3 | (TAT)4 | 12 | 45786 | 45797 |
|  | 22 | c | (A)10(AT)5 | 72 | 45960 | 46031 |
|  | 23 | p1 | (T)12 | 12 | 49736 | 49747 |
|  | 24 | p1 | (T)10 | 10 | 53321 | 53330 |
|  | 25 | p1 | (T)12 | 12 | 58640 | 58651 |
|  | 26 | p3 | (ATA)4 | 12 | 64209 | 64220 |
|  | 27 | p1 | (T)11 | 11 | 65273 | 65283 |
|  | 28 | p4 | (AAAC)3 | 12 | 66888 | 66899 |
|  | 29 | p1 | (A)10 | 10 | 68860 | 68869 |
|  | 30 | p1 | (T)11 | 11 | 70236 | 70246 |
|  | 31 | p2 | (AT)7 | 14 | 75115 | 75128 |
|  | 32 | p1 | (T)10 | 10 | 80814 | 80823 |
|  | 33 | p3 | (TAT)4 | 12 | 81012 | 81023 |
|  | 34 | p2 | (TA)7 | 14 | 82604 | 82617 |
|  | 35 | p3 | (TAT)4 | 12 | 84866 | 84877 |
|  | 36 | p3 | (TGA)4 | 12 | 90125 | 90136 |
|  | 37 | p4 | (TTTA)4 | 16 | 106566 | 106581 |
|  | 38 | p1 | (T)10 | 10 | 110440 | 110449 |
|  | 39 | p4 | (AATA)3 | 12 | 112789 | 112800 |
|  | 40 | p1 | (A)10 | 10 | 114170 | 114179 |
|  | 41 | p1 | (G)11 | 11 | 115865 | 115875 |
|  | 42 | p4 | (AATC)3 | 12 | 117973 | 117984 |
|  | 43 | p1 | (A)11 | 11 | 118142 | 118152 |
|  | 44 | p3 | (ATA)4 | 12 | 120701 | 120712 |
|  | 45 | p1 | (T)11 | 11 | 123192 | 123202 |
|  | 46 | p1 | (T)10 | 10 | 123483 | 123492 |
|  | 47 | p1 | (T)10 | 10 | 123667 | 123676 |
|  | 48 | p1 | (T)12 | 12 | 124052 | 124063 |
|  | 49 | p3 | (TCT)5 | 15 | 124457 | 124471 |
|  | 50 | p1 | (T)11 | 11 | 125238 | 125248 |
|  | 51 | p4 | (AATA)4 | 16 | 127002 | 127017 |
|  | 52 | p3 | (ATC)4 | 12 | 143448 | 143459 |
|  | 53 | p3 | (ATA)4 | 12 | 148708 | 148719 |
| *D. peruviana* | 1 | p3 | (AAT)4 | 12 | 4220 | 4231 |
|  | 2 | p4 | (ATTG)3 | 12 | 5507 | 5518 |
|  | 3 | p2 | (AT)6 | 12 | 7334 | 7345 |
|  | 4 | p1 | (T)10 | 10 | 7478 | 7487 |
|  | 5 | p1 | (A)11 | 11 | 7699 | 7709 |
|  | 6 | p1 | (T)10 | 10 | 8379 | 8388 |
|  | 7 | c | (A)10(A)10 | 48 | 9355 | 9402 |
|  | 8 | p1 | (T)13 | 13 | 9563 | 9575 |
|  | 9 | p1 | (A)11 | 11 | 11765 | 11775 |
|  | 10 | p1 | (A)10 | 10 | 14624 | 14633 |
|  | 11 | c | (A)12(A)10 | 43 | 15572 | 15614 |
|  | 12 | p2 | (TA)5 | 10 | 19179 | 19188 |
|  | 13 | p2 | (AT)5 | 10 | 20210 | 20219 |
|  | 14 | p2 | (AT)9 | 18 | 30505 | 30522 |
|  | 15 | p1 | (T)12 | 12 | 31442 | 31453 |
|  | 16 | p3 | (TTC)4 | 12 | 34292 | 34303 |
|  | 17 | c | (T)10(G)11 | 21 | 35378 | 35398 |
|  | 18 | p1 | (A)10 | 10 | 41479 | 41488 |
|  | 19 | p1 | (T)10 | 10 | 42423 | 42432 |
|  | 20 | p1 | (T)10 | 10 | 45159 | 45168 |
|  | 21 | c | (ATAA)3(TA)7 | 64 | 45412 | 45475 |
|  | 22 | p3 | (TAT)4 | 12 | 45937 | 45948 |
|  | 23 | p2 | (AT)5 | 10 | 46172 | 46181 |
|  | 24 | p1 | (T)11 | 11 | 49886 | 49896 |
|  | 25 | p1 | (T)10 | 10 | 53464 | 53473 |
|  | 26 | p1 | (T)11 | 11 | 58783 | 58793 |
|  | 27 | p3 | (ATA)4 | 12 | 64351 | 64362 |
|  | 28 | p1 | (T)10 | 10 | 65415 | 65424 |
|  | 29 | p4 | (AAAC)3 | 12 | 67029 | 67040 |
|  | 30 | p1 | (A)10 | 10 | 69001 | 69010 |
|  | 31 | p1 | (T)10 | 10 | 70377 | 70386 |
|  | 32 | p2 | (AT)7 | 14 | 75248 | 75261 |
|  | 33 | p1 | (T)10 | 10 | 80947 | 80956 |
|  | 34 | p3 | (TAT)4 | 12 | 81193 | 81204 |
|  | 35 | p2 | (TA)6 | 12 | 82785 | 82796 |
|  | 36 | p3 | (TAT)4 | 12 | 85045 | 85056 |
|  | 37 | p3 | (TGA)4 | 12 | 90304 | 90315 |
|  | 38 | p4 | (TTTA)4 | 16 | 106745 | 106760 |
|  | 39 | p1 | (T)10 | 10 | 110619 | 110628 |
|  | 40 | p4 | (AATA)3 | 12 | 112968 | 112979 |
|  | 41 | p1 | (A)11 | 11 | 114349 | 114359 |
|  | 42 | p4 | (AATC)3 | 12 | 118151 | 118162 |
|  | 43 | p1 | (A)10 | 10 | 118320 | 118329 |
|  | 44 | p3 | (ATA)4 | 12 | 120878 | 120889 |
|  | 45 | p1 | (T)11 | 11 | 123369 | 123379 |
|  | 46 | p1 | (T)10 | 10 | 123660 | 123669 |
|  | 47 | p1 | (T)10 | 10 | 123844 | 123853 |
|  | 48 | p1 | (T)12 | 12 | 124229 | 124240 |
|  | 49 | p3 | (TCT)5 | 15 | 124634 | 124648 |
|  | 50 | p1 | (T)11 | 11 | 125415 | 125425 |
|  | 51 | p4 | (AATA)4 | 16 | 127179 | 127194 |
|  | 52 | p3 | (ATC)4 | 12 | 143625 | 143636 |
|  | 53 | p3 | (ATA)4 | 12 | 148885 | 148896 |
| *D. montana* | 1 | p3 | (AAT)4 | 12 | 4116 | 4127 |
|  | 2 | p4 | (ATTG)3 | 12 | 5403 | 5414 |
|  | 3 | p2 | (AT)6 | 12 | 7230 | 7241 |
|  | 4 | p1 | (T)10 | 10 | 7374 | 7383 |
|  | 5 | p1 | (A)10 | 10 | 7595 | 7604 |
|  | 6 | p1 | (T)10 | 10 | 8274 | 8283 |
|  | 7 | p1 | (A)10 | 10 | 9250 | 9259 |
|  | 8 | p1 | (T)14 | 14 | 9457 | 9470 |
|  | 9 | c | (A)11(T)10 | 92 | 11660 | 11751 |
|  | 10 | p1 | (A)10 | 10 | 14520 | 14529 |
|  | 11 | c | (A)13(A)10 | 44 | 15468 | 15511 |
|  | 12 | p2 | (TA)5 | 10 | 19076 | 19085 |
|  | 13 | p2 | (AT)5 | 10 | 20107 | 20116 |
|  | 14 | p1 | (T)12 | 12 | 31292 | 31303 |
|  | 15 | p3 | (TTC)4 | 12 | 34142 | 34153 |
|  | 16 | c | (T)12(G)12 | 24 | 35228 | 35251 |
|  | 17 | p1 | (A)10 | 10 | 41332 | 41341 |
|  | 18 | p1 | (T)10 | 10 | 42276 | 42285 |
|  | 19 | p1 | (T)10 | 10 | 45012 | 45021 |
|  | 20 | c | (ATAA)3(TA)7 | 64 | 45265 | 45328 |
|  | 21 | p3 | (TAT)4 | 12 | 45790 | 45801 |
|  | 22 | c | (A)10(AT)5 | 72 | 45964 | 46035 |
|  | 23 | p1 | (T)13 | 13 | 49740 | 49752 |
|  | 24 | p1 | (T)10 | 10 | 53326 | 53335 |
|  | 25 | p1 | (T)11 | 11 | 58645 | 58655 |
|  | 26 | p3 | (ATA)4 | 12 | 64185 | 64196 |
|  | 27 | p1 | (T)11 | 11 | 65248 | 65258 |
|  | 28 | p4 | (AAAC)3 | 12 | 66863 | 66874 |
|  | 29 | p1 | (A)10 | 10 | 68815 | 68824 |
|  | 30 | p1 | (T)10 | 10 | 70191 | 70200 |
|  | 31 | p2 | (AT)7 | 14 | 75069 | 75082 |
|  | 32 | p1 | (T)10 | 10 | 80768 | 80777 |
|  | 33 | p3 | (TAT)4 | 12 | 80966 | 80977 |
|  | 34 | p2 | (TA)6 | 12 | 82558 | 82569 |
|  | 35 | p3 | (TAT)4 | 12 | 84818 | 84829 |
|  | 36 | p3 | (TGA)4 | 12 | 90077 | 90088 |
|  | 37 | p4 | (TTTA)4 | 16 | 106518 | 106533 |
|  | 38 | p1 | (T)10 | 10 | 110392 | 110401 |
|  | 39 | p4 | (AATA)3 | 12 | 112741 | 112752 |
|  | 40 | p1 | (A)10 | 10 | 114122 | 114131 |
|  | 41 | p1 | (G)11 | 11 | 115817 | 115827 |
|  | 42 | p4 | (AATC)3 | 12 | 117925 | 117936 |
|  | 43 | p1 | (A)10 | 10 | 118094 | 118103 |
|  | 44 | p3 | (ATA)4 | 12 | 120652 | 120663 |
|  | 45 | p1 | (T)11 | 11 | 123143 | 123153 |
|  | 46 | p1 | (T)10 | 10 | 123434 | 123443 |
|  | 47 | p1 | (T)10 | 10 | 123618 | 123627 |
|  | 48 | p1 | (T)12 | 12 | 124003 | 124014 |
|  | 49 | p3 | (TCT)5 | 15 | 124408 | 124422 |
|  | 50 | p1 | (T)11 | 11 | 125189 | 125199 |
|  | 51 | p4 | (AATA)4 | 16 | 126953 | 126968 |
|  | 52 | p3 | (ATC)4 | 12 | 143399 | 143410 |
|  | 53 | p3 | (ATA)4 | 12 | 148659 | 148670 |
| *D. ruiziana* | 1 | p3 | (AAT)4 | 12 | 4107 | 4118 |
|  | 2 | p4 | (ATTG)3 | 12 | 5394 | 5405 |
|  | 3 | p4 | (ACTT)3 | 12 | 6707 | 6718 |
|  | 4 | p2 | (AT)6 | 12 | 7222 | 7233 |
|  | 5 | p1 | (T)10 | 10 | 7366 | 7375 |
|  | 6 | p1 | (A)11 | 11 | 7587 | 7597 |
|  | 7 | p1 | (T)10 | 10 | 8267 | 8276 |
|  | 8 | p1 | (A)10 | 10 | 9243 | 9252 |
|  | 9 | p1 | (T)13 | 13 | 9450 | 9462 |
|  | 10 | c | (A)10(T)10 | 91 | 11652 | 11742 |
|  | 11 | c | (A)13(A)11 | 45 | 15458 | 15502 |
|  | 12 | p2 | (TA)5 | 10 | 19067 | 19076 |
|  | 13 | p2 | (AT)5 | 10 | 20098 | 20107 |
|  | 14 | p1 | (T)12 | 12 | 31266 | 31277 |
|  | 15 | p3 | (TTC)4 | 12 | 34116 | 34127 |
|  | 16 | p1 | (T)16 | 16 | 35202 | 35217 |
|  | 17 | p1 | (A)10 | 10 | 41307 | 41316 |
|  | 18 | p1 | (T)10 | 10 | 42251 | 42260 |
|  | 19 | p1 | (T)10 | 10 | 44987 | 44996 |
|  | 20 | c | (ATAA)6(TA)7 | 76 | 45240 | 45315 |
|  | 21 | p3 | (TAT)4 | 12 | 45777 | 45788 |
|  | 22 | c | (A)10(AT)5 | 72 | 45951 | 46022 |
|  | 23 | p1 | (T)13 | 13 | 49727 | 49739 |
|  | 24 | p1 | (T)10 | 10 | 53313 | 53322 |
|  | 25 | p1 | (T)12 | 12 | 58640 | 58651 |
|  | 26 | p3 | (ATA)4 | 12 | 64209 | 64220 |
|  | 27 | p1 | (T)11 | 11 | 65273 | 65283 |
|  | 28 | p4 | (AAAC)3 | 12 | 66888 | 66899 |
|  | 29 | p1 | (A)11 | 11 | 68861 | 68871 |
|  | 30 | p1 | (T)10 | 10 | 70238 | 70247 |
|  | 31 | p2 | (AT)7 | 14 | 75116 | 75129 |
|  | 32 | p1 | (T)10 | 10 | 80815 | 80824 |
|  | 33 | p3 | (TAT)4 | 12 | 81013 | 81024 |
|  | 34 | p2 | (TA)6 | 12 | 82605 | 82616 |
|  | 35 | p3 | (TAT)4 | 12 | 84872 | 84883 |
|  | 36 | p3 | (TGA)4 | 12 | 90131 | 90142 |
|  | 37 | p4 | (TTTA)4 | 16 | 106572 | 106587 |
|  | 38 | p1 | (T)10 | 10 | 110446 | 110455 |
|  | 39 | p4 | (AATA)3 | 12 | 112795 | 112806 |
|  | 40 | p1 | (A)10 | 10 | 114176 | 114185 |
|  | 41 | p1 | (G)11 | 11 | 115871 | 115881 |
|  | 42 | p4 | (AATC)3 | 12 | 117979 | 117990 |
|  | 43 | p1 | (A)10 | 10 | 118148 | 118157 |
|  | 44 | p3 | (ATA)4 | 12 | 120706 | 120717 |
|  | 45 | p1 | (T)11 | 11 | 123197 | 123207 |
|  | 46 | p1 | (T)10 | 10 | 123488 | 123497 |
|  | 47 | p1 | (T)10 | 10 | 123672 | 123681 |
|  | 48 | p1 | (T)12 | 12 | 124057 | 124068 |
|  | 49 | p3 | (TCT)5 | 15 | 124462 | 124476 |
|  | 50 | p1 | (T)11 | 11 | 125243 | 125253 |
|  | 51 | p4 | (AATA)4 | 16 | 127007 | 127022 |
|  | 52 | p3 | (ATC)4 | 12 | 143453 | 143464 |
|  | 53 | p3 | (ATA)4 | 12 | 148713 | 148724 |
| *D. mucronata* | 1 | p3 | (AAT)4 | 12 | 4220 | 4231 |
|  | 2 | p4 | (ATTG)3 | 12 | 5507 | 5518 |
|  | 3 | p2 | (AT)6 | 12 | 7335 | 7346 |
|  | 4 | p1 | (T)10 | 10 | 7479 | 7488 |
|  | 5 | p1 | (A)13 | 13 | 7700 | 7712 |
|  | 6 | p1 | (T)10 | 10 | 8382 | 8391 |
|  | 7 | p1 | (A)10 | 10 | 9358 | 9367 |
|  | 8 | p1 | (T)13 | 13 | 9559 | 9571 |
|  | 9 | c | (A)11(T)10 | 92 | 11761 | 11852 |
|  | 10 | c | (A)13(A)11 | 45 | 15568 | 15612 |
|  | 11 | p2 | (TA)5 | 10 | 19177 | 19186 |
|  | 12 | p2 | (AT)5 | 10 | 20208 | 20217 |
|  | 13 | p2 | (TA)8 | 16 | 30474 | 30489 |
|  | 14 | p1 | (T)12 | 12 | 31392 | 31403 |
|  | 15 | p3 | (TTC)4 | 12 | 34242 | 34253 |
|  | 16 | p1 | (T)14 | 14 | 35328 | 35341 |
|  | 17 | p1 | (A)10 | 10 | 41430 | 41439 |
|  | 18 | p1 | (T)10 | 10 | 42374 | 42383 |
|  | 19 | p1 | (T)11 | 11 | 45110 | 45120 |
|  | 20 | c | (ATAA)3(TA)7 | 64 | 45364 | 45427 |
|  | 21 | p3 | (TAT)4 | 12 | 45889 | 45900 |
|  | 22 | c | (A)10(AT)5 | 72 | 46063 | 46134 |
|  | 23 | p1 | (T)11 | 11 | 49839 | 49849 |
|  | 24 | p1 | (T)10 | 10 | 53423 | 53432 |
|  | 25 | p1 | (T)11 | 11 | 58742 | 58752 |
|  | 26 | p3 | (ATA)4 | 12 | 64310 | 64321 |
|  | 27 | p1 | (T)10 | 10 | 65374 | 65383 |
|  | 28 | p4 | (AAAC)3 | 12 | 66988 | 66999 |
|  | 29 | p1 | (A)10 | 10 | 68940 | 68949 |
|  | 30 | p2 | (AT)7 | 14 | 75194 | 75207 |
|  | 31 | p1 | (T)10 | 10 | 80894 | 80903 |
|  | 32 | p3 | (TAT)4 | 12 | 81108 | 81119 |
|  | 33 | p2 | (TA)6 | 12 | 82700 | 82711 |
|  | 34 | p3 | (TAT)4 | 12 | 84960 | 84971 |
|  | 35 | p3 | (TGA)4 | 12 | 90219 | 90230 |
|  | 36 | p4 | (TTTA)4 | 16 | 106660 | 106675 |
|  | 37 | p1 | (T)11 | 11 | 110528 | 110538 |
|  | 38 | p4 | (AATA)3 | 12 | 112878 | 112889 |
|  | 39 | p1 | (A)10 | 10 | 114259 | 114268 |
|  | 40 | p1 | (G)10 | 10 | 115954 | 115963 |
|  | 41 | p4 | (AATC)3 | 12 | 118061 | 118072 |
|  | 42 | p3 | (ATA)4 | 12 | 120787 | 120798 |
|  | 43 | p1 | (T)11 | 11 | 123278 | 123288 |
|  | 44 | p1 | (T)10 | 10 | 123569 | 123578 |
|  | 45 | p1 | (T)10 | 10 | 123753 | 123762 |
|  | 46 | p1 | (T)12 | 12 | 124138 | 124149 |
|  | 47 | p3 | (TCT)5 | 15 | 124543 | 124557 |
|  | 48 | p1 | (T)11 | 11 | 125324 | 125334 |
|  | 49 | p4 | (AATA)4 | 16 | 127088 | 127103 |
|  | 50 | p3 | (ATC)4 | 12 | 143534 | 143545 |
|  | 51 | p3 | (ATA)4 | 12 | 148794 | 148805 |
