# Supplementary material for: Comparative analysis of chloroplast genomes for five Dicliptera species (Acanthaceae): molecular structure, phylogenetic relationships, and adaptive evolution
Source: PeerJ. 2020 Feb 6;8:e8450. doi: 10.7717/peerj.8450 (PMC7007973; doi:10.7717/peerj.8450)
Supplement: Table S6 [file peerj-08-8450-s006.docx]

**Table S6.** Long repeat sequences in the cp genomes of the five *Dicliptera* species.

| **Species** | **Size of repeat Ⅰ** | **Repeat start Ⅰ** | **Match direction** | **Size of repeat Ⅱ** | **Repeat start Ⅱ** | **Distance of repeat** | **E-value** |
| --- | --- | --- | --- | --- | --- | --- | --- |
| *D. acuminata* | 41 | 97044 | F | 41 | 117255 | 0 | 1.32E-15 |
|  | 41 | 117255 | P | 41 | 136499 | 0 | 1.32E-15 |
|  | 39 | 42567 | F | 39 | 97046 | 0 | 2.11E-14 |
|  | 39 | 42567 | F | 39 | 117257 | 0 | 2.11E-14 |
|  | 39 | 42567 | P | 39 | 136499 | 0 | 2.11E-14 |
|  | 41 | 37613 | F | 41 | 39837 | -3 | 3.80E-10 |
|  | 30 | 7626 | P | 30 | 44038 | 0 | 5.54E-09 |
|  | 31 | 7622 | F | 31 | 34501 | -3 | 1.68E-04 |
|  | 31 | 7743 | F | 31 | 9154 | -3 | 1.68E-04 |
|  | 30 | 9026 | P | 30 | 94020 | -3 | 6.08E-04 |
|  | 30 | 9026 | F | 30 | 139534 | -3 | 6.08E-04 |
|  | 30 | 9107 | F | 30 | 35433 | -3 | 6.08E-04 |
|  | 30 | 87730 | F | 30 | 87772 | -3 | 6.08E-04 |
|  | 30 | 87730 | P | 30 | 145782 | -3 | 6.08E-04 |
|  | 30 | 87772 | P | 30 | 145824 | -3 | 6.08E-04 |
|  | 30 | 145782 | F | 30 | 145824 | -3 | 6.08E-04 |
| *D. peruviana* | 49 | 81007 | F | 49 | 81023 | 0 | 2.02E-20 |
|  | 41 | 97223 | F | 41 | 117433 | 0 | 1.32E-15 |
|  | 41 | 117433 | P | 41 | 136676 | 0 | 1.32E-15 |
|  | 39 | 42724 | F | 39 | 97225 | 0 | 2.12E-14 |
|  | 39 | 42724 | F | 39 | 117435 | 0 | 2.12E-14 |
|  | 39 | 42724 | P | 39 | 136676 | 0 | 2.12E-14 |
|  | 33 | 81007 | F | 33 | 81039 | 0 | 8.67E-11 |
|  | 41 | 37770 | F | 41 | 39994 | -3 | 3.81E-10 |
|  | 30 | 7730 | P | 30 | 44195 | 0 | 5.55E-09 |
|  | 37 | 30967 | R | 37 | 30977 | -3 | 7.10E-08 |
|  | 32 | 30475 | P | 32 | 30485 | -2 | 1.55E-06 |
|  | 31 | 7726 | F | 31 | 34661 | -3 | 1.68E-04 |
|  | 31 | 7847 | F | 31 | 9258 | -3 | 1.68E-04 |
|  | 30 | 9130 | P | 30 | 94199 | -3 | 6.08E-04 |
|  | 30 | 9130 | F | 30 | 139711 | -3 | 6.08E-04 |
|  | 30 | 9211 | F | 30 | 35590 | -3 | 6.08E-04 |
|  | 30 | 30492 | C | 30 | 30493 | -3 | 6.08E-04 |
|  | 30 | 30963 | F | 30 | 30987 | -3 | 6.08E-04 |
|  | 30 | 45453 | C | 30 | 45454 | -3 | 6.08E-04 |
|  | 30 | 87909 | F | 30 | 87951 | -3 | 6.08E-04 |
|  | 30 | 87909 | P | 30 | 145959 | -3 | 6.08E-04 |
|  | 30 | 87951 | P | 30 | 146001 | -3 | 6.08E-04 |
|  | 30 | 145959 | F | 30 | 146001 | -3 | 6.08E-04 |
| *D. montana* | 41 | 96996 | F | 41 | 117207 | 0 | 1.32E-15 |
|  | 41 | 117207 | P | 41 | 136450 | 0 | 1.32E-15 |
|  | 39 | 42577 | F | 39 | 96998 | 0 | 2.11E-14 |
|  | 39 | 42577 | F | 39 | 117209 | 0 | 2.11E-14 |
|  | 39 | 42577 | P | 39 | 136450 | 0 | 2.11E-14 |
|  | 41 | 37623 | F | 41 | 39847 | -3 | 3.80E-10 |
|  | 30 | 7625 | P | 30 | 44048 | 0 | 5.54E-09 |
|  | 31 | 7621 | F | 31 | 34511 | -3 | 1.68E-04 |
|  | 31 | 7742 | F | 31 | 9153 | -3 | 1.68E-04 |
|  | 30 | 9025 | P | 30 | 93972 | -3 | 6.07E-04 |
|  | 30 | 9025 | F | 30 | 139485 | -3 | 6.07E-04 |
|  | 30 | 9106 | F | 30 | 35443 | -3 | 6.07E-04 |
|  | 30 | 45306 | C | 30 | 45307 | -3 | 6.07E-04 |
|  | 30 | 87682 | F | 30 | 87724 | -3 | 6.07E-04 |
|  | 30 | 87682 | P | 30 | 145733 | -3 | 6.07E-04 |
|  | 30 | 87724 | P | 30 | 145775 | -3 | 6.07E-04 |
|  | 30 | 145733 | F | 30 | 145775 | -3 | 6.07E-04 |
| *D. ruiziana* | 41 | 97050 | F | 41 | 117261 | 0 | 1.32E-15 |
|  | 41 | 117261 | P | 41 | 136504 | 0 | 1.32E-15 |
|  | 39 | 42552 | F | 39 | 97052 | 0 | 2.11E-14 |
|  | 39 | 42552 | F | 39 | 117263 | 0 | 2.11E-14 |
|  | 39 | 42552 | P | 39 | 136504 | 0 | 2.11E-14 |
|  | 41 | 37598 | F | 41 | 39822 | -3 | 3.80E-10 |
|  | 30 | 7618 | P | 30 | 44023 | 0 | 5.54E-09 |
|  | 30 | 45237 | R | 30 | 45238 | -1 | 4.99E-07 |
|  | 31 | 7614 | F | 31 | 34485 | -3 | 1.68E-04 |
|  | 31 | 7735 | F | 31 | 9146 | -3 | 1.68E-04 |
|  | 30 | 9018 | P | 30 | 94026 | -3 | 6.08E-04 |
|  | 30 | 9018 | F | 30 | 139539 | -3 | 6.08E-04 |
|  | 30 | 9099 | F | 30 | 35418 | -3 | 6.08E-04 |
|  | 30 | 45293 | C | 30 | 45294 | -3 | 6.08E-04 |
|  | 30 | 87736 | F | 30 | 87778 | -3 | 6.08E-04 |
|  | 30 | 87736 | P | 30 | 145787 | -3 | 6.08E-04 |
|  | 30 | 87778 | P | 30 | 145829 | -3 | 6.08E-04 |
|  | 30 | 145787 | F | 30 | 145829 | -3 | 6.08E-04 |
| *D. mucronata* | 41 | 97138 | F | 41 | 117343 | 0 | 1.32E-15 |
|  | 41 | 117343 | P | 41 | 136585 | 0 | 1.32E-15 |
|  | 39 | 42675 | F | 39 | 97140 | 0 | 2.11E-14 |
|  | 39 | 42675 | F | 39 | 117345 | 0 | 2.11E-14 |
|  | 39 | 42675 | P | 39 | 136585 | 0 | 2.11E-14 |
|  | 41 | 37721 | F | 41 | 39945 | -3 | 3.80E-10 |
|  | 30 | 7733 | P | 30 | 44146 | 0 | 5.54E-09 |
|  | 31 | 7729 | F | 31 | 34611 | -3 | 1.68E-04 |
|  | 31 | 7850 | F | 31 | 9261 | -3 | 1.68E-04 |
|  | 31 | 30474 | C | 31 | 45405 | -3 | 1.68E-04 |
|  | 30 | 9133 | P | 30 | 94114 | -3 | 6.07E-04 |
|  | 30 | 9133 | F | 30 | 139620 | -3 | 6.07E-04 |
|  | 30 | 9214 | F | 30 | 35541 | -3 | 6.07E-04 |
|  | 30 | 45405 | C | 30 | 45406 | -3 | 6.07E-04 |
|  | 30 | 87824 | F | 30 | 87866 | -3 | 6.07E-04 |
|  | 30 | 87824 | P | 30 | 145868 | -3 | 6.07E-04 |
|  | 30 | 87866 | P | 30 | 145910 | -3 | 6.07E-04 |
|  | 30 | 145868 | F | 30 | 145910 | -3 | 6.07E-04 |
