# Supplementary material for: Comparative analysis of chloroplast genomes for five Dicliptera species (Acanthaceae): molecular structure, phylogenetic relationships, and adaptive evolution
Source: PeerJ. 2020 Feb 6;8:e8450. doi: 10.7717/peerj.8450 (PMC7007973; doi:10.7717/peerj.8450)
Supplement: Table S7 [file peerj-08-8450-s007.docx]

**Table S7.** Summary of long repeats and simple sequence repeats (SSRs).

| **long repeats** | | | | | | | **Simple sequence repeats (SSR)** | | | | |
| --- | --- | --- | --- | --- | --- | --- | --- | --- | --- | --- | --- |
| **species** | **Tandem repeats** | **Complement repeats** | **Forward repeats** | **Palindromic repeats** | **Reverse repeats** | **Total** | **Mononucleotide** | **Dinucleotides** | **Trinucleotides** | **Tetranucleotides** | **Total** |
| *D. acuminata* | 25 | 2 | 10 | 7 | 0 | 44 | 33 | 7 | 11 | 7 | 58 |
| *D. peruviana* | 31 | 2 | 13 | 9 | 1 | 56 | 31 | 8 | 11 | 7 | 57 |
| *D. montana* | 23 | 1 | 10 | 7 | 0 | 41 | 33 | 7 | 11 | 7 | 58 |
| *D. ruiziana* | 23 | 1 | 10 | 7 | 1 | 42 | 31 | 7 | 11 | 8 | 57 |
| *D. mucronata* | 26 | 2 | 10 | 8 | 0 | 46 | 29 | 8 | 11 | 7 | 55 |
| **Sum** | 128 | 8 | 53 | 38 | 2 | 229 | 157 | 37 | 55 | 36 | 285 |
| **Percentage** | 0.5590 | 0.0349 | 0.2314 | 0.1659 | 0.0087 |  | 0.5509 | 0.1298 | 0.1930 | 0.1263 |  |
