# Supplementary material for: Comparative analysis of chloroplast genomes for five Dicliptera species (Acanthaceae): molecular structure, phylogenetic relationships, and adaptive evolution
Source: PeerJ. 2020 Feb 6;8:e8450. doi: 10.7717/peerj.8450 (PMC7007973; doi:10.7717/peerj.8450)
Supplement: Table S8 [file peerj-08-8450-s008.docx]

**Table S8.** Non-synonymous (K_A_) and synonymous (K_S_) substitution rates and ω value (ω=K_A_/K_S_) of orthologous gene sequence.

| **gene** | **K_A_** | **K_S_** | ω=**K_A_/K_S_** |
| --- | --- | --- | --- |
| *accD* | 0.1723 | 0.5518 | 0.31224 |
| *atpA* | 0.0605 | 0.4469 | 0.13527 |
| *atpB* | 0.0262 | 0.3785 | 0.06923 |
| *atpE* | 0.0987 | 0.3419 | 0.28859 |
| *atpF* | 0.1334 | 0.5499 | 0.24261 |
| *atpH* | 0 | 0.4162 | 0.0001 |
| *atpI* | 0.0419 | 0.4728 | 0.08859 |
| *ccsA* | 0.2546 | 0.7095 | 0.35883 |
| *cemA* | 0.1187 | 0.3474 | 0.34175 |
| *clpP* | 0.1052 | 0.3609 | 0.29149 |
| *infA* | 0.0231 | 0.4331 | 0.05342 |
| *matK* | 0.334 | 0.3547 | 0.94182 |
| *ndhA* | 0.0919 | 0.6003 | 0.15305 |
| *ndhB* | 0.0108 | 0.0547 | 0.19702 |
| *ndhC* | 0.0722 | 0.3261 | 0.22135 |
| *ndhD* | 0.1317 | 0.6355 | 0.20726 |
| *ndhE* | 0.073 | 0.4551 | 0.16032 |
| *ndhF* | 0.2515 | 1.0079 | 0.24955 |
| *ndhG* | 0.0989 | 0.4033 | 0.24522 |
| *ndhH* | 0.0561 | 0.6548 | 0.0857 |
| *ndhI* | 0.0567 | 0.5873 | 0.09657 |
| *ndhJ* | 0.0807 | 0.5058 | 0.1595 |
| *ndhK* | 0.0245 | 0.318 | 0.07715 |
| *petA* | 0.0878 | 0.4634 | 0.18953 |
| *petB* | 0.0232 | 0.4111 | 0.05634 |
| *petD* | 0.0086 | 0.6329 | 0.01358 |
| *petG* | 0.0281 | 0.2239 | 0.12539 |
| *petL* | 0.0586 | 0.536 | 0.10931 |
| *petN* | 0.0165 | 0.2742 | 0.06002 |
| *psaA* | 0.0221 | 0.3746 | 0.05894 |
| *psaB* | 0.0126 | 0.4065 | 0.03088 |
| *psaC* | 0.0112 | 0.5497 | 0.02042 |
| *psaI* | 0.0982 | 0.7015 | 0.13996 |
| *psaJ* | 0.0115 | 0.396 | 0.02915 |
| *psbA* | 0.0142 | 0.3676 | 0.0385 |
| *psbB* | 0.0154 | 0.4576 | 0.03374 |
| *psbC* | 0.0184 | 0.3342 | 0.05491 |
| *psbD* | 0.0104 | 0.3259 | 0.0318 |
| *psbE* | 0.0105 | 0.301 | 0.03477 |
| *psbF* | 0 | 0.0742 | 0.0001 |
| *psbH* | 0.0304 | 0.4591 | 0.06631 |
| *psbI* | 0.0127 | 0.7412 | 0.0172 |
| *psbJ* | 0.0333 | 0.0789 | 0.42176 |
| *psbK* | 0.0891 | 0.4085 | 0.21814 |
| *psbL* | 0.013 | 0.2219 | 0.05852 |
| *psbM* | 0.0129 | 0.1258 | 0.10285 |
| *psbN* | 0.0462 | 0.2419 | 0.19117 |
| *psbT* | 0.0136 | 0.4928 | 0.02768 |
| *psbZ* | 0.1142 | 0.311 | 0.36701 |
| *rbcL* | 0.0527 | 0.406 | 0.12972 |
| *rp33* | 0.1284 | 0.3463 | 0.3707 |
| *rp36* | 0.0484 | 0.3933 | 0.12313 |
| *rpl14* | 0.0794 | 0.4924 | 0.16134 |
| *rpl16* | 0.044 | 0.6101 | 0.07206 |
| *rpl20* | 0.1613 | 0.3481 | 0.46329 |
| *rpl23* | 0.0898 | 0.2073 | 0.43302 |
| *rpoA* | 0.1523 | 0.4086 | 0.37274 |
| *rpoB* | 0.0757 | 0.3683 | 0.20567 |
| *rps14* | 0.0565 | 0.3338 | 0.16925 |
| *rps15* | 0.2125 | 0.4943 | 0.42987 |
| *rps16* | 0.1519 | 0.6139 | 0.24742 |
| *rps18* | 0.0895 | 0.3669 | 0.24378 |
| *rps3* | 0.1017 | 0.6513 | 0.15614 |
| *rps4* | 0.0825 | 0.3718 | 0.22203 |
| *rps8* | 0.1074 | 0.5532 | 0.19418 |
| *yc4* | 0.0954 | 0.4148 | 0.23013 |
| *ycf15* | 0.0708 | 0.049 | 1.44528 |
| *ycf3* | 0.0208 | 0.4314 | 0.04816 |
